# Roles of Multiple Globus Pallidus Territories of Monkeys and Humans in Motivation, Cognition and Action: An Anatomical, Physiological and Pathophysiological Review

**DOI:** 10.3389/fnana.2017.00030

**Published:** 2017-04-10

**Authors:** Yosuke Saga, Eiji Hoshi, Léon Tremblay

**Affiliations:** ^1^Institute of Cognitive Science Marc Jeannerod, UMR-5229 CNRSBron, France; ^2^Frontal Lobe Function Project, Tokyo Metropolitan Institute of Medical ScienceTokyo, Japan; ^3^AMED-CREST, Japan Agency for Medical Research and DevelopmentTokyo, Japan

**Keywords:** globus pallidus, nonhuman primate, human, functional territory, GABA, cortico-basal ganglia circuit, rabies virus

## Abstract

The globus pallidus (GP) communicates with widespread cortical areas that support various functions, including motivation, cognition and action. Anatomical tract-tracing studies revealed that the anteroventral GP communicates with the medial prefrontal and orbitofrontal cortices, which are involved in motivational control; the anterodorsal GP communicates with the lateral prefrontal cortex, which is involved in cognitive control; and the posterior GP communicates with the frontal motor cortex, which is involved in action control. This organization suggests that distinct subdivisions within the GP play specific roles. Neurophysiological studies examining GP neurons in monkeys during behavior revealed that the types of information coding performed within these subdivisions differ greatly. The anteroventral GP is characterized by activities related to motivation, such as reward seeking and aversive avoidance; the anterodorsal GP is characterized by activity that reflects cognition, such as goal decision and action selection; and the posterior GP is characterized by activity associated with action preparation and execution. Pathophysiological studies have shown that GABA-related substances or GP lesions result in abnormal activity in the GP, which causes site-specific behavioral and motor symptoms. The present review article discusses the anatomical organization, physiology and pathophysiology of the three major GP territories in nonhuman primates and humans.

## Introduction

The globus pallidus (GP) is a principal component of the basal ganglia (BG) and can be divided into the external segment (GPe, intermediate nucleus) and the internal segment (GPi, output nucleus). Both of these GP nuclei receive major afferents from the striatum, which is the main input station of the BG, and the subthalamic nucleus (STN), which mediates the indirect pathway of the BG and also constitutes an input station (Parent et al., 1984, 1995; Haber et al., 1990, 1993, 1995; Flaherty and Graybiel, 1993; Kaneda et al., 2002; François et al., 2004). The major output targets of the GPe include the GPi, substantia nigra pars reticulata (SNr) and STN (Parent, 1990) while the GPi projects to the thalamus, which relays these signals to the cerebral cortex (Kim et al., 1976; Wiesendanger and Wiesendanger, 1985; Matelli et al., 1989; Matelli and Luppino, 1996; Sakai et al., 1996). Additionally, GPe neurons project back to the striatum to modulate the activity of striatal neurons (Staines et al., 1981; Beckstead, 1983; Jayaraman, 1983; Bevan et al., 1998; Kita et al., 1999; Bolam et al., 2000). Therefore, the GPe is a key node involved in the control of information flow through the BG, whereas the GPi integrates converging signals from cortical areas (via the striatum), the STN and the GPe to give rise to output signals to the cerebral cortex via the thalamus (Percheron et al., 1984; Bolam et al., 2000).

Many studies have used conventional anterograde and retrograde tracers to map the anatomical networks of cortico-BG circuits and revealed patterns of connectivity between structures that possess direct connections. Alexander et al. (1986) reviewed a large number of these anatomical studies and described five cortico-BG circuits: the motor, oculomotor, dorsolateral prefrontal, lateral orbitofrontal and anterior cingulate circuits. However, because cortico-BG circuits are composed of multiple synapses, novel techniques were required to determine the detailed organization of these networks. Subsequently, the transneuronal tract-tracing method using a neurotropic virus was determined to be the most suitable technique for this type of investigation, because these viruses selectively infect neurons and are then transported across synapses in a time-dependent manner (Ugolini et al., 1989; Zemanick et al., 1991; Kelly and Strick, 2000). Studies using this technique identified finer connectivity patterns between specific cortical areas, such as projections from specific sub-regions of the BG to the motor and association cortices (Middleton and Strick, 1994, 2000b, 2002; Kelly and Strick, 2004; Akkal et al., 2007; Saga et al., 2011).

In agreement with the anatomical studies, functional studies have revealed that distinct frontal cortical areas play central roles in a variety of functions, including motivation, action and cognition (Miller and Cohen, 2001; Cisek, 2007; Tanji and Hoshi, 2008). Findings also revealed that dysfunction within the BG causes a variety of behavioral deficits in action, cognition and motivation. To provide a better understanding of the roles that the cortico-BG networks play under conditions of health and disease, the present review article will discuss the findings of anatomical, physiological and pathophysiological studies investigating the function of the GP in nonhuman primates and humans. Additionally, this review article will describe multiple anatomical territories of the GP and the manner in which each contributes to deficits in motor, cognitive and motivational functions as well as pathological disorders induced by their dysfunction.

## The Nonhuman Primate GP Is Composed of Three Territories

**Figure 1:**
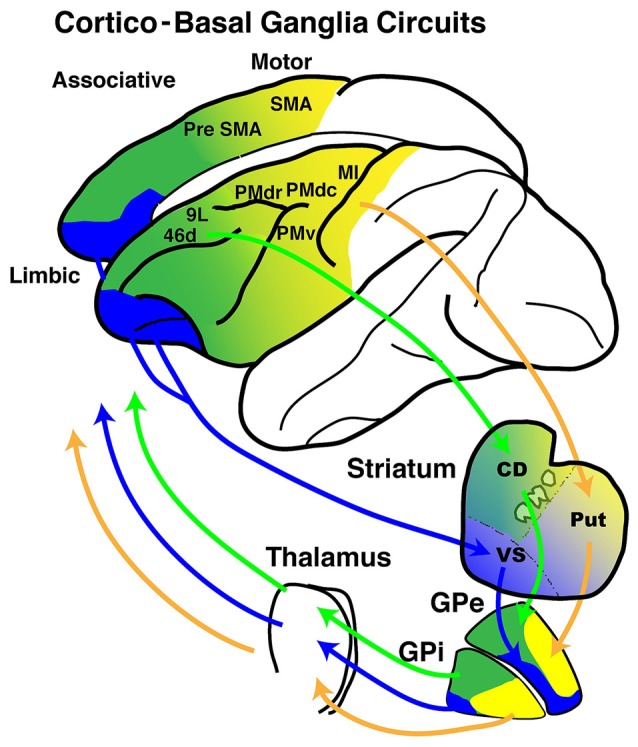
**Schematic diagrams of cortico- basal ganglia (BG) anatomical loops with functional territories.** The motor (yellow), associative (green) and limbic (blue) territories are represented in circuits between the frontal cortical areas and the BG.

Anatomical studies have provided major contributions to the present understanding of the neuronal circuits that connect the frontal cortical areas and the BG (Figure 1). In particular, the transneuronal transport of viruses such as herpes simplex virus type 1(HSV-1) and the rabies virus (RV) has revealed connections across synapses (Kelly and Strick, 2000; Dum and Strick, 2013) because these viruses infect neurons and are then transported across synapses in a time-dependent manner. More specifically, the injection of neurotropic viruses into cortical areas has illustrated that specific portions of the GPi and GPe send either disynaptic (from the GPi) or trisynaptic (from the GPe) projections to cortical areas.

**Figure 2:**
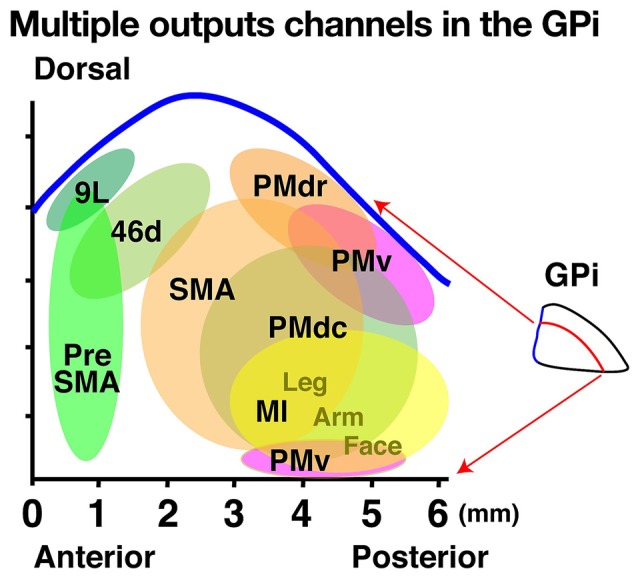
**Multiple output channels in the internal segment of the globus pallidus (GPi) projecting to cortical areas represented on a two-dimensional unfolded map.** The horizontal and vertical axes indicate the anteroposterior and dorsoventral axes, respectively. Each colored circle in the map represents the output territory of the GPi confirmed by rabies virus (RV) studies. Tick mark intervals = 1 mm.

For example, Hoover and Strick (1993) injected HSV-1 into the arm regions of the supplementary motor area (SMA), primary motor cortex (MI) and ventral premotor cortex (PMv) in monkeys. They demonstrated that labeled GPi neurons projecting to these areas are distributed in distinct subportions of the middle-to-posterior aspect of the nucleus with the SMA-projecting neurons located most dorsally, PMv-projecting neurons located most ventrally, and MI-projecting neurons located in between these regions. Thus, this landmark study revealed that distinct subdivisions of the GPi send outputs to distinct frontal cortical areas. Subsequently, these authors examined the somatotopic organizations of the MI-projecting region by injecting either HSV-1 or RV into the arm, leg, or face regions of the MI (Hoover and Strick, 1999). Similar to their previous study, labeled GPi neurons were identified in the posterior and ventral aspects of the nucleus (see Figure 2). Additionally, they identified a somatotopic organization within the MI-projecting region such that GPi neurons projecting to the MI leg region are located in the rostrodorsal portion, those projecting to the MI face region are in the caudoventral portion, and those projecting to the MI arm region are in between. However, the overall distributions of neurons projecting to the distinct body parts or to different areas are not completely segregated. These results indicate that there is an MI-subdivision in the posterior aspect of the GP, and that this area has a somatotopic organization with partial overlaps of different body parts.

To reveal the GPi subportions that project to higher-order motor areas, Akkal et al. (2007) injected RV into the SMA and pre-SMA and identified labeled GPi neurons in distinct portions of this region (Figure 2). After the SMA injections, labeled neurons were found in the central portion of the GPi, while labeled neurons were evident in the anterior portion of the GPi after the pre-SMA injections (Figure 2). Thus, the GPi subportions that project to the SMA and pre-SMA differ from each other, and both of these regions differ from the subportion that projects to the MI.

Subsequently, we investigated the GP areas that send outputs to the dorsal PM (PMd) by separately injecting RV into the anterior and ventral aspects of the PMd (Saga et al., 2011). We found labeled neurons in the middle dorsal portion of the GPi and determined that the anterior and posterior portions of the PMd (PMdr and PMdc, respectively) receive main inputs from distinct subportions of the GPi. The PMdr receives main inputs from the more anterior aspect of the middle dorsal portion of the GPi while the PMdc receives inputs from the more posterior aspect. To identify the GPi subportion that sends outputs to the PMv, Ishida et al. (2016) injected RV into this region and found that the labeled neurons were mainly located in the dorsal and most posterior parts of the GPi. Thus, the middle to posterior part of the dorsal GPi sends outputs to the PM and distinct subportions of the GPi send outputs to specific premotor areas.

Much like the GPi regions that send outputs to the motor and higher-order motor areas, other regions, including the prefrontal cortex (Brodmann areas 46, 9 and 12), also send outputs to the association cortex (Middleton and Strick, 1994, 2000a, 2002). Following injections into either area 46 or 9, labeled neurons were primarily observed in the anterodorsal portion of the GPi, which is largely different from the GP portions that send outputs to the motor areas (Hoover and Strick, 1993). In contrast, injections into areas 46v and 12l resulted in only a few labeled neurons in the GPi but revealed that the SNr is a main output area to this cortical region (Middleton and Strick, 2002). These studies successfully identified a specific subportion of the GPi (the anterodorsal aspect) that sends outputs to the prefrontal cortex. Viral tracing studies such as these rarely result in labeling of the anteroventral aspect of the GP, but conventional tracers have shown that this region of the GP receives major inputs from the ventral striatum and the ventral aspects of other BG nuclei (Spooren et al., 1996; François et al., 2004; Haber and Knutson, 2010; Sgambato-Faure et al., 2016). These connectivity patterns suggest that the anteroventral GP mainly communicates with limbic areas in the cerebral cortex.

Taken together, these findings indicate that the GPi is composed of three major territories: the motor territory, the association territory and the limbic territory. The relative localization of the three territories are described in terms of anterior-posterior and dorsal-ventral axes, however, this does not simply refer to the anterior/posterior or dorsal/ventral halves. In this article, we use these terms to indicate a relative localization or a trend. The motor territory is located in the posterior part of the GPi, while the associative territory is located in the anterodorsal part and the limbic territory is located in the anteroventral part. Moreover, although there are partial overlaps, each territory can be further divided into sub-regions according to cortical target areas.

As with the GPi, the neuronal circuits of the GPe have been investigated using neurotropic viruses. Injection of a virus into the MI results in neuronal labeling in the posteroventral portion of the GPe (Kelly and Strick, 2004). In contrast, viral injections into the prefrontal cortex result in neuronal labeling in the anterodorsal portion of the GPe (Kelly and Strick, 2004), which indicates that this subportion corresponds to the associative territory. Because no studies to date have injected viral injections into the limbic cortex, the location of the limbic territory within the GPe remains unclear. The anteroventral aspect of the GP is shown to receive main inputs from the anteroventral striatum (Haber et al., 1985; Spooren et al., 1996), which suggests that this portion of the GPe corresponds to the limbic territory. Within the limbic territory, the subdivision below the anterior commissure is known as the ventral pallidum (VP; Alheid and Heimer, 1988).

In contrast to the GPi labeling, the viral injections into the PMdc and PMv result in broader neuronal labeling in the both dorsal and ventral portions of the posterior GPe and the anterior portion corresponding to the association territory (Saga et al., 2011; Ishida et al., 2016). This broader labeling of GPe neurons suggests that the three territories may be less segregated within the GPe.

Taken together, these anatomical studies demonstrate that both the GPe and GPi possess motor, associative and limbic territories within each nucleus however, the mode of the segregation may differ in the GPi and GPe. Additionally, both the GPi and GPe have output channels directed to the motor cortex, prefrontal cortex and limbic cortex, and each of these output channels receives main inputs from the targeted cortical areas (Haber et al., 1990, 1993, 1995, 2006; Hedreen and DeLong, 1991; Kunishio and Haber, 1994; François et al., 2004; Calzavara et al., 2007; Worbe et al., 2012; Sgambato-Faure et al., 2016). This anatomical organization suggests that the GP processes information from diverse cortical areas and, more specifically, that there may also be functional territories composed of motor, association and limbic regions within the GPi and GPe.

## The Three Territories of the Human GP as Revealed by Imaging Studies

In humans, diffusion tensor imaging (DTI) studies have shown that the association, motor and limbic cortices project to distinct portions of the striatum (Lehéricy et al., 2004). The association cortex projects to the anterodorsal portion, the motor and PM project to the posterior portion, and the limbic cortex to the anteroventral portion. Additional imaging studies support the existence of segregated territories within the human striatum (Di Martino et al., 2008; Choi et al., 2012; Jarbo and Verstynen, 2015). Taken together, these findings indicate that the overall projection pattern of the human GP is consistent with that of the nonhuman primate GP (Draganski et al., 2008; Averbeck et al., 2014).

**Figure 3:**
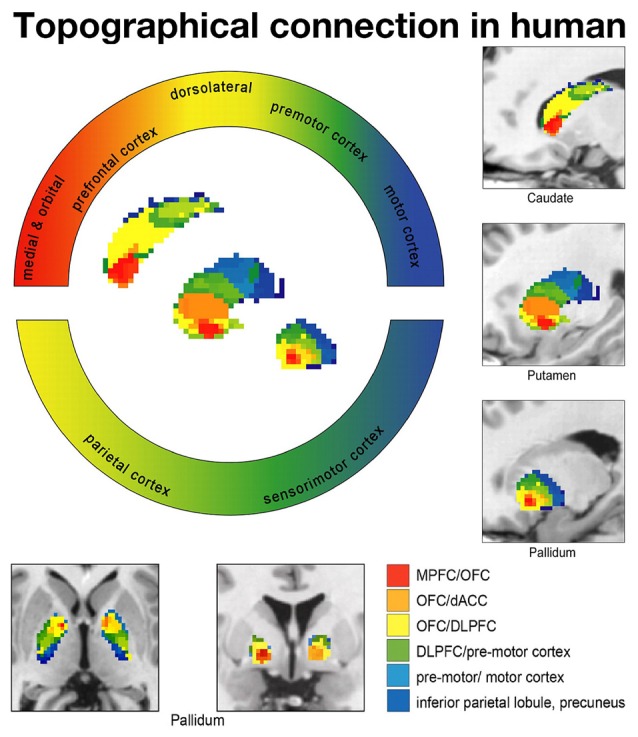
**Diffusion tensor imaging (DTI) study showing topographical connections between the cortex and BG in humans.** Each frontal cortical area projects to different territories in the BG. Limbic (red: medial and orbital frontal cortices), associative (yellow: dorsolateral prefrontal cortex), higher motor (green: premotor cortex) and motor (blue: motor cortex) cortical territories project to corresponding territories in the BG according to a topographical distribution. Data from Draganski et al. (2008) used with permission from the Society for Neuroscience.

Draganski et al. (2008) investigated the projection patterns from the cerebral cortex to the GP (Figure 3). They found that the prefrontal cortex, motor cortex and the limbic cortex project to the anterodorsal (associative territory), posteroventral portion (motor territory) and anteroventral portion of the GP (limbic territory), respectively. Thus, these tract imaging studies demonstrated that the human GP is largely composed of the associative, motor and limbic territories and that the topographical organization is similar to that of monkeys.

## The Motor, Associative and Motivational Territories in the GP of Nonhuman Primates

**Figure 4:**
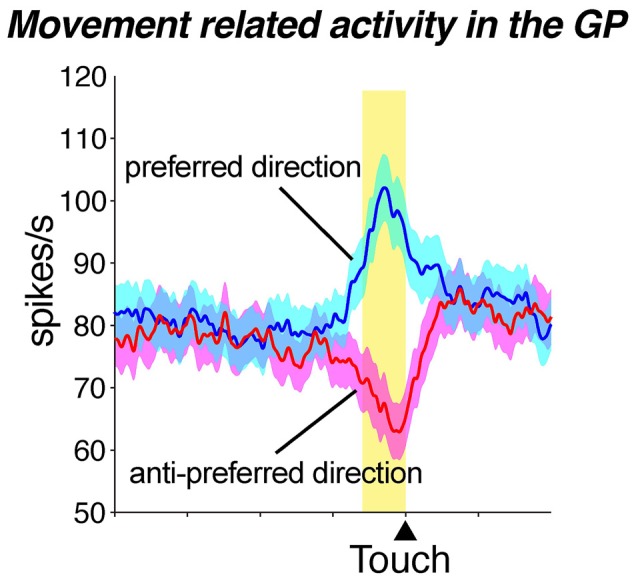
**Movement-related activity exhibiting directional preferences in the globus pallidus (GP).** Movement-related population activity for the preferred direction (blue) and the anti-preferred direction (red: mean ± SEM) showing excitation and inhibition, respectively. The yellow zone represents the average time of the movement period and the histogram is aligned with touch the screen (“Touch”). Data from Saga et al. (2013) used with permission from the Society for Neuroscience.

**Figure 5:**
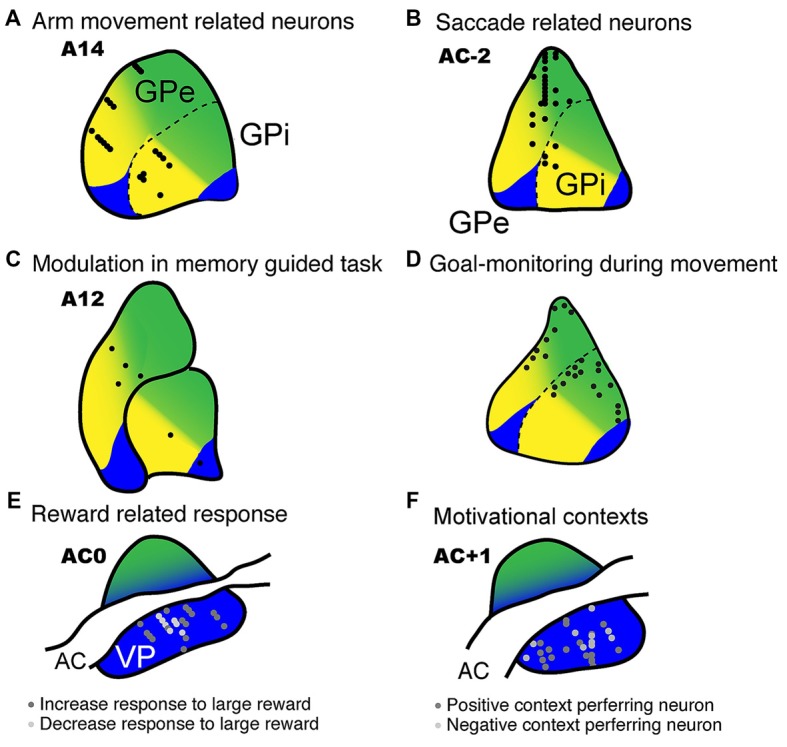
**Electrophysiological recordings from the GP with estimated functional territories.** Neuronal activity recorded from the GP revealed task-related neurons during a variety of task conditions. **(A)** Movement-related activity mainly found in the ventral aspect of the GP (DeLong, 1971; data modified and used with the American Physiological Society). **(B)** Anti-saccade related neurons recorded in the dorsal aspect of the GP (Yoshida and Tanaka, 2009; data used with permission from Oxford University Press). **(C)** Recording sites of neuronal activity modulated by memory-guided reaching movements (Turner and Anderson, 2005; data modified and used with permission from the Society for Neuroscience). **(D)** Goal-monitoring neurons identified in the dorsal to middle aspect of the GP during movement in which monkeys monitor the behavioral goal (spatial or object, Saga et al., 2013; data modified and used with permission from the Society for Neuroscience). **(E)** Reward-related neurons in the ventral pallidum (VP), which also show reward expectation activity for larger rewards (Tachibana and Hikosaka, 2012; data modified and used with permission from Elsevier). **(F)** VP neurons showing positive or negative context preference (Saga et al., 2016; data modified and used with permission from Oxford University Press).

Given that three different territories exist within the GP, it can be expected that each territory plays a specific role. Electrophysiological studies conducted during the performance of a variety of behaviors have elucidated the functions of the GP, as well as the cerebral cortex and other brain areas. DeLong (1971) was the first to record neuronal activity in the GP of behaving monkeys and reported that GP neurons show high spontaneous activity, are characterized by either tonic or burst activity, and exhibit excitatory and inhibitory responses in relation to multiple behavioral events. A subset of GP neurons primarily located in the middle to ventral portion of the posterior GP shows movement-related activity with direction selectivity (Figure 4); this region corresponds to the motor territory that has been anatomically defined (Figure 5A). Subsequent studies identified GP neurons that exhibit movement-related activity influenced by behavioral contexts, such as a movement sequence (Mushiake and Strick, 1995) or the absence or presence of visual guidance (Figure 5C; Turner and Anderson, 1997, 2005). Dysfunction within the GP motor territory induced by microinjections of muscimol (GABA_A_ receptor agonist) results in abnormal posture (Horak and Anderson, 1984; Mink and Thach, 1991; Desmurget and Turner, 2008), impairments in the execution of movements (such as reaching velocity and acceleration), and hypometria (Turner and Desmurget, 2010). These results further substantiate the notion that the anatomically defined motor territory plays a crucial role in the execution of movement.

GP neurons in the associative territory respond to the appearance of visual signals and the presentation of cues that instruct future actions or signal reward probability (Figure 5B; Arkadir et al., 2004; Pasquereau et al., 2007; Yoshida and Tanaka, 2009; Arimura et al., 2013). Arimura et al. (2013) showed that GP neurons in the associative territory encode behavioral goals (reaching to a relatively left- or rightward target) as well as actions (actual location of the target on a screen), indicating that this region is involved in goal-directed decisions and action selection. Furthermore, GP neurons monitor behavioral goal (Figure 5D, spatial or object). The striatal region, which is thought to project to the GP and head and body of the caudate nucleus, plays a major role in learning stimulus-response associations (Pasupathy and Miller, 2005). Moreover, high frequency electrical microstimulation (200 Hz) in the anterior caudate, which projects to the associative territory of the GP, but not to the putamen (which projects to the motor territory), improves the performance of monkeys in a visual reward learning task (Williams and Eskandar, 2006). Taken together, these results indicate that the associative territory of the GP is involved in the cognitive aspects of action control, such as decision-making and action selection, rather than motor execution *per se*.

In addition to the motor and cognitive functions described above, the GP in monkeys is involved in motivational functions such as processing appetitive and aversive signals. When monkeys perform a reward-based saccade task, neurons in the VP, which is a subportion of the GPe ventral to the anterior commissure, exhibit reward expectation and reward-related responses (Figure 5E; Tachibana and Hikosaka, 2012). Furthermore, VP neurons exhibit excitatory responses following the appearance of a visual target that is indicative of a larger reward, but show inhibitory responses to a target indicative of a small reward. In that study, the latencies of the saccadic eye movements in the monkeys were typically shorter during large reward trials than during small reward trials, which suggests that oculomotor behavior is biased by the degree of the expected reward. On the other hand, inactivation of the bilateral VP by microinjections of muscimol results in the loss of biased behaviors. Taken together, these results indicate that the VP plays a crucial role in positively motivated behavior.

**Figure 6:**
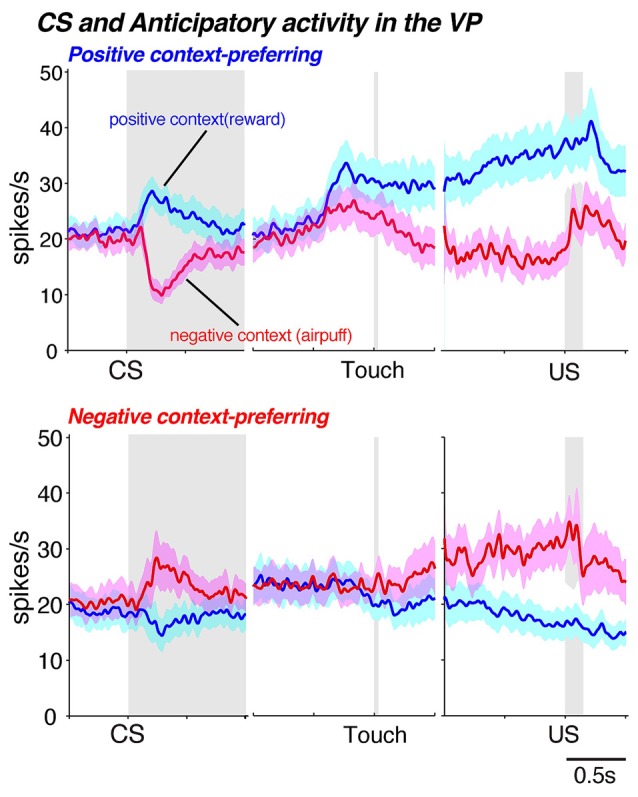
**Neuronal population activities for positive motivational context-preferring (upper panel) and negative motivational context-preferring neurons (lower panel) in the VP for conditioned stimulus (CS)-related activity and anticipatory activity.** Monkeys performed different motivational tasks in which they chose approach or avoidance behaviors to obtain a reward or prevent the delivery of an air puff depending on the motivational context (Saga et al., 2016). The blue histogram (mean ± SEM) indicates neuronal activity in trials in the positive motivational context and red represents activity in trials in the negative motivational context. Each panel is aligned with CS onset, touch screen and unconditioned stimulus (US) delivery and the gray zone represents the time period of the behavioral event. Data modified from Saga et al. (2016) used with permission from Oxford University Press.

In a specific Pavlovian conditioning task, neurons in the GPi and GPe responded to the appearance of a visual stimulus that was associated with either reward (juice) or aversion (air puff to the face) as well as to the delivery of the outcome (reward or air puff, Joshua et al., 2009). Thus, the GP is involved in processing not only appetitive signals but aversive signals as well. We further investigated this phenomenon as monkeys performed a behavioral task and found that the VP is involved in negatively as well as positively motivated behavior (Saga et al., 2016). In that particular task, a conditioned stimulus (CS) predicted either an appetitive outcome (a drop of juice) or an aversive outcome (air puff) and the monkeys were required to choose between approaching or avoiding the cue by performing a reaching movement. Usually, the monkeys made an approach behavior in the appetitive context and an avoidance behavior in the aversive context. These context dependent behaviors indicate that the CSs provide the information concerning motivational context. Accordingly, the VP neurons of these monkeys discriminated between the appetitive and aversive contexts (or between positive and negative motivations) by exhibiting excitatory and inhibitory activity modulation during the presentation of the CS (Figures 5F, 6). Additionally, the VP neurons showed anticipatory activity in advance of the delivery of the outcome. In another study, excessive activation of the VP caused by microinjections of bicuculline (a GABA_A_ antagonist) resulted in decreased avoidance behavior in the aversive context, as well as the premature termination of a trial (Saga et al., 2016). Interestingly, this effect was observed exclusively in the aversive motivational context, which suggests that excessive activation of the VP increases sensitivity to aversive contexts. Excessive activation of the VP is known to result in repetitive licking and biting of the fingers (Grabli et al., 2004), which is a behavioral hallmark of anxiety or stress. Thus, these results indicate that VP plays a crucial role in the performance of behaviors in the aversive as well as appetitive context.

In sum, there are functional segregations within the GP of monkeys that are in line with functional connectivity observed in anatomical studies. The posterior part of the GP, which belongs to the motor network, is involved in motor functions; the anterodorsal part of the GP, which belongs to the associative network, is involved in cognitive functions; and the rostroventral aspect of the GP (VP), which belongs to the limbic network, is involved in motivational functions.

## The Motor, Cognitive and Motivational Functions of the Human GP

Similar to the nonhuman primate GP, involvement of the human GP in motor function has been widely substantiated. Functional brain imaging studies revealed activation in the GP in relation to a variety of motor acts, including sequential movements, precision grip and the manipulation of a joystick (Boecker et al., 1998; Turner et al., 1998, 2003; Vaillancourt et al., 2004). Furthermore, these studies showed that activation signals in the GP are correlated with the velocity and amplitude of a movement and there are functional differences between the GPi and GPe. Although the production of an identical amplitude during a precision grip results in comparable activation in the GPi and GPe, stronger activation is observed in the GPe relative to the GPi when the subject is required to choose between different amplitudes (Vaillancourt et al., 2007). Thus, the GPe is involved in the selection of movements. A recent report reviewed evidence regarding whether the GPe and GPi play distinct roles and determined that the anterior aspects of the BG (the caudate, putamen and GPe) are involved in planning, while the posterior nuclei of the BG (the posterior putamen and GPi) are related to the movement parameters of precision grip (Prodoehl et al., 2009).

With respect to motor function, other studies identified activation in the GP during sequential movements (Boecker et al., 1998; Hanakawa et al., 2008). For example, Lehéricy et al. (2006) compared brain activity during a complex sequential movement (a sequence of 10 finger movements) with activity that occurred during a simple movement (repetitive flexion movement of the index finger). They found stronger activation in sensory motor areas, the parietal cortex, premotor areas and the prefrontal cortex during the execution of the complex movement relative to the simple movement. Additionally, these authors reported that the complex movement results in enhanced activation of the anterior aspects of the GP (associative territory) and striatum, while the simple movement results in enhanced activation of the posterior aspects of the GP (motor territory) and striatum; this corresponds with the findings of a previous study (Boecker et al., 1998). These differential activation patterns indicate that the anterior part of the GP is involved in executing complex movements or cognitive functions associated with motor execution in conjunction with the association cortex, which is similar to the prefrontal cortex. On the other hand, the posterior part of the GP is involved in the execution of movement in conjunction with the MI, which is similar to the PM.

Previous research has revealed that the GP is also involved in cognitive functions. For example, Corbetta et al. (1991) examined the role of the human GP during an attention processing task that incorporated visual stimuli. The dorsal GP (the associative territory linking to caudate nucleus) was activated when subjects detected a change in visual stimulus features (i.e., speed, color and/or shape), which indicates that the GP is involved in visual attention. In addition to the associative territories of the GP and BG, the prefrontal cortex was also found to be essential for filtering attention during the storage of relevant information, which is an association function, in a short-term memory task (McNab and Klingberg, 2008). Furthermore, Aron and Poldrack (2006) found that the GP and the STN are activated when subjects inhibit prepared action. Taken together, these findings suggest that the GP is involved in higher cognitive functions, such as attention and action inhibition.

In addition to motor and cognitive functions, the human GP is also involved in motivational function. A functional magnetic resonance imaging (fMRI) study investigated the neural representations of incentive motivation and the acquisition of a monetary reward using visual stimuli that predicted different monetary rewards that could be obtained if a lever was gripped strongly enough (Pessiglione et al., 2007); a second visual cue instructed on the extent of the force that was to be generated. These authors found that activity in the VP reflected a future reward during the presentation of the stimuli; i.e., incentive motivation. Subsequently, they asked patients suffering from an auto-activation deficit (AAD) induced by bi-pallidal lesion, which leads to general dysfunction in the GP, to perform this task (Schmidt et al., 2008). Although the AAD patients could perform the grip movements according to the instructions, they made fewer attempts to obtain the indicated monetary rewards (loss of motivation); the authors proposed that VP dysfunction resulted in this deficit.

Similar to negative motivation contexts in monkeys, the human GP is activated by the presentation of disgust pictures (Phillips et al., 1998) and activity in the VP in response to such pictures is correlated with activation in the anterior insula (Calder et al., 2007). Because electrical stimulation of the ventral part of the anterior insula leads to disgust-related behavior (vomiting and food rejection) in human and nonhuman primates, the VP may receive negative reward signals from the anterior insula (Catenoix et al., 2008; Caruana et al., 2011; Jezzini et al., 2012). Accordingly, anatomical studies have shown that the VP receives inputs from the anterior insula via the ventral striatum (Spooren et al., 1996; Chikama et al., 1997). Taken together, these results indicate that the anterior aspect of the human GP, particularly the VP, is involved in opposite functions, such as negative and positive motivation.

## Deficits in Motor, Cognitive and Motivational Functions Induced by Dysfunction in the GP in Nonhuman Primates

Parkinson’s disease is one of the most common disorders resulting from dysfunction within the BG. An animal model of Parkinson’s disease utilizes injections of 1-methyl-4-phenyl-1,2,3,6-tetrahydropyridine (MPTP), which destroys dopaminergic neurons in the midbrain and causes dysfunction in the BG due to the lack of dopaminergic innervation (Stern, 1990). The GP neurons in MPTP-treated monkeys lose functional selectivity and begin to respond to electrical stimulation in a much wider area of the striatum (Tremblay et al., 1989), which suggests that this dysfunction involves the GP. Additionally, MPTP-treated monkeys exhibit various motor symptoms (Tremblay et al., 2015), and there is a correlation between the level of dopamine in the GP and parkinsonian motor score (Ballanger et al., 2016). Moreover, the GP is involved in compensatory mechanisms (Neumane et al., 2012). In MPTP-treated monkeys, dopamine loss occurs not only in the motor territory (posterior striatum) but also in the associative territory (anterodorsal striatum), which is related to dysfunction of cognitive aspects (Jan et al., 2003; Pessiglione et al., 2004). Taken together, these findings indicate that the MPTP animal model results in functional deficits within multiple territories of the GP and striatum.

Clinical pathological conditions are thought to be due to either hyperactivity or hypoactivity within a specific territory of the GP. Accordingly, several animal models have been developed to induce a variety of symptoms that allow for the investigation of relationships between neuronal activity under normal conditions and the behavioral symptoms induced by abnormal activity in the same regions. These models provide causal evidence that GP neurons directly contribute to changes in behavior under conditions of both health and disease.

Injections of GABA-receptor related substances have been used to induce hypoactivity or hyperactivity in specific GP territories. For example, GABA_A_-receptor agonists, such as muscimol, and GABA_A_-receptor antagonists, such as bicuculline and gabagine, are widely used to induce reversible hypoactivity and hyperactivity, respectively. Lesions or injection of muscimol into the posterior aspect of the GPi (motor territory) cause reductions in the velocity and peak acceleration of reaching movements and alters the trajectories of movements, which is known as hypometria (Mink and Thach, 1991; Kato and Kimura, 1992; Inase et al., 1996; Wenger et al., 1999; Desmurget and Turner, 2008). However, these motor symptoms do not appear to be generalized, as the direction of movement, the reaction times and the patterns of muscle activation were preserved. Additionally, the execution of learned sequential movements following inactivation of the GPi motor territory are comparable to those observed prior to the injection (Turner and Desmurget, 2010). Thus, the GP motor territory seems to contribute to specific components of movement, such as the generation of velocity and amplitude, which is consistent with the notion that the GP is involved in the generation of movement vigor (Desmurget and Turner, 2008).

To investigate the impact of hyperactivation within the GPe, Grabli et al. (2004) independently injected bicuculline into the anterodorsal (associative), posterior (motor) and anteroventral (limbic) territories of the GP and reported distinct effects. Hyperactivity in the motor territory (posterior GPe) results in abnormal movements; hyperactivity in the associative territory (anterodorsal GP) results in attention deficits, hyperactivity, and circling to the side contralateral to the injected hemisphere; and hyperactivity in the limbic territory (the VP or anteroventral GPe) results in repetitive finger biting and licking (compulsive behaviors). Hypoactivity in the VP results in attenuation of positively motivated behavior (Tachibana and Hikosaka, 2012), as well as enhanced sensitivity within aversive contexts (Saga et al., 2016). Although they investigated a region outside the VP, Piron et al. (2016) demonstrated that bilateral injections of muscimol into the center of the GPi impair the association learning of cues and reward probabilities, but not of well-learned associations between cues and reward probability. These findings indicate that the learning process may involve, and require functionality within, multiple functional territories. Accordingly, Nixon et al. (2004) found that lesions within networks linking the GP and PMd cause deficits in association learning, which suggests that the GP plays an important role in this process.

In addition to expressing receptors for GABA-related substances, GP neurons express receptors for glutamate (Kita et al., 2004; Tachibana et al., 2008), dopamine, serotonin (Sgambato-Faure and Tremblay, 2017) and acetylcholine (Eid and Parent, 2016). Thus, it will be necessary to investigate the contributions of these neurotransmitters to various GP functions.

## Motor and Non-Motor Deficits Induced by Dysfunction in the Human GP

Clinical symptoms indicate that dysfunction within the human BG is responsible, at least in part, for movement disorders (DeLong and Wichmann, 2007). Similar to MPTP-treated monkeys, patients with Parkinson’s disease exhibit motor symptoms and non-motor symptoms (NMS), such as cognitive impairments (Chaudhuri et al., 2006; Chaudhuri and Schapira, 2009). As observed in MPTP-parkinsonian monkeys, dopaminergic degeneration occurs in both the motor and associative territories of the striatum (Brooks and Piccini, 2006; Tremblay et al., 2015). Moreover, depression, which is another NMS associated with BG dysfunction, is observed in patients with Parkinson’s disease (Chaudhuri et al., 2006). This type of depression is thought to be the result of altered function in the limbic territory because its severity is inversely correlated with dopamine availability in this region (Remy et al., 2005). Deep brain stimulation (DBS) of the GPi and STN effectively treats a variety of parkinsonian movement symptoms, as well as multiple types of NMS (Fasano et al., 2012). Thus, it appears that the relationships between degeneration in the BG and motor and NMS are in accordance with the concept of functional territories within the GP and BG.

Lesions of subcortical areas, including the striatum and pallidum, also induce NMS (Ward et al., 2013) and subjects with BG damage exhibit impaired performances in the delayed matching-to-sample task, which requires short-term memory (Voytek and Knight, 2010). In addition to altering cognitive processes, dysfunction in the BG disturbs motivated behaviors and can result in the loss of motivation (abulia, Dubois et al., 1995; Benke et al., 2003; Pulsipher et al., 2006). Laplane et al. (1984) found that lesions of the human GP result in an apathetic state and, although the patients in this study were able to move as instructed, they rarely initiated action voluntarily. Furthermore, both GP lesions and pallidotomies induce a transient manic state (Starkstein et al., 1990; Okun et al., 2003a). When a pallidotomy is performed in the anteromedial part of the GPi, patients show manic behaviors such as increased energy, manifested in performing excessive amounts of housework, and not requiring sleep (Okun et al., 2003a). These specific lesion effects indicate that the functional territories of the human GP are consistent with those of nonhuman primates.

If distinct territories are responsible for distinct functions, neuropsychiatric syndromes, such as anxiety-related disorders, obsessive-compulsive disorder (OCD), Tourette’s syndrome, and major depressive disorder, may be treated by modulating activities within specific territories of the GP or BG using DBS or pharmacological substances (Malone et al., 2009; Krack et al., 2010; Williams and Okun, 2013). However, some clinical studies have reported that DBS is associated with adverse side effects, including anxiety (Okun et al., 2003b; Shapira et al., 2006; Williams and Okun, 2013). Nonetheless, a greater understanding of the organization of the human GP and BG is necessary to safely administer clinical therapies aimed at treating the various symptoms caused by dysfunction within the GP or BG. Preclinical investigations in non-human primates are warranted to determine the most relevant section of the GP to target for the stimulation, similar to the methods used to determine the involvement of the STN in compulsive behaviors (Baup et al., 2008).

## Conclusions

In monkeys, the GP belongs to multiple functional circuits that network through the BG and frontal cortical areas. Like the frontal cortex, the GP of monkeys is composed of multiple functional territories that support association, motor, and motivational functions. Anatomical, physiological and pathophysiological studies in nonhuman primates indicate that distinct subportions of the GP play distinct roles in behavior and current clinical evidence suggests that the overall structural and functional architectures of these regions are similar in humans and monkeys. Most importantly, dysfunctions within specific subportions of the GP appear to be responsible for specific neurological and psychiatric disorders. However, further studies are needed to reveal the precise architecture of the GP and BG in both humans and monkeys, which in turn will lead to a better understanding of GP and BG functions and improved treatment options for the symptoms induced by these neurological and psychiatric disorders.

## Author Contributions

YS, EH and LT wrote the manuscript.

## Conflict of Interest Statement

The authors declare that the research was conducted in the absence of any commercial or financial relationships that could be construed as a potential conflict of interest.
